# Case Report: novel GUCA1B and ABHD12 mutations in retinitis pigmentosa sine pigmento: expanding the genotypic spectrum through multimodal phenotyping

**DOI:** 10.3389/fmed.2025.1622343

**Published:** 2025-10-20

**Authors:** Yujie Wu, Haochen Wang, Jingkai Zhang, Xiufang Wang, Xiaohan Wu, Chunjie Mao, Yajie Sun, Wei Zhou

**Affiliations:** ^1^Department of Ophthalmology, Tianjin Medical University General Hospital, Tianjin, China; ^2^Department of Operating Theatre, Tianjin Medical University General Hospital, Tianjin, China; ^3^Department of Ophthalmology, Tianjin Medical University General Hospital, Konggang Hospital, Tianjin, China

**Keywords:** retinitis pigmentosa sine pigmento, GUCA1B, ABHD12, novel mutation, multimodal imaging, calcium signaling, lipid metabolism

## Abstract

Retinitis pigmentosa sine pigmento (RPSP) is a atypical variant of inherited retinal degeneration characterized by the absence of retinal pigment deposits observed in typical retinitis pigmentosa, which poses significant challenges to clinical diagnosis and genetic investigation. Although high-throughput sequencing technologies have revolutionized the identification of disease-causing genes, studies on RPSP are limited. Therefore, this study aimed to analyze the clinical manifestations and genetic profiles of two patients with RPSP. Two novel potential disease-causing mutations were identified. Patient 1 had a heterozygous missense mutation (c.45G > C, p.Glu15Asp) in the GUCA1B gene, whereas Patient 2 had a homozygous frameshift insertion mutation (c.134_137dupCGGC, p.Ala47Glyfs*4) in the ABHD12 gene. Multimodal imaging techniques, including optical coherence tomography, fundus autofluorescence, adaptive optics scanning laser ophthalmoscope, and fluorescein angiography, combined with visual electrophysiological assessments revealed the structural and functional retinal alterations associated with RPSP. Bioinformatics analysis revealed that these mutations can respectively contributed to disease development by affecting calcium ion regulation in photoreceptor cells and by influencing the hydrolyzing of lysophosphatidylserine (lyso-PS). This study is the first to link novel mutations in GUCA1B and ABHD12 to RPSP. The findings highlight the critical importance of integrating multimodal imaging with genetic profiling in enhancing early diagnostic accuracy and refining genetic counseling strategies for this understudied condition.

## Introduction

Retinitis pigmentosa (RP) is an inherited retinal disorder characterized by progressive degeneration of photoreceptors and retinal pigment epithelium (RPE) (1, 2). The global prevalence of RP ranges from 1 in 3,500 individuals to 1 in 5,000 individuals (3). A 2024 nationwide screening in China involving nearly two million participants reported a prevalence of 0.35 per 1,000 (95% CI: 0.31–0.40) (4). RP is also recognized as a leading cause of visual impairment in young and working-age individuals, underscoring its significant clinical and societal impact, subtypes include classic RP sine pigmento (RPSP), crystalline RP (Bietti’s crystalline dystrophy), and paravenous RP (5). RP is characterized by marked genetic and phenotypic heterogeneity, with more than 90 disease-associated genes identified to date (3). However, causative mutations have not been identified in 30%–40% of cases (6).

Retinitis pigmentosa sine pigmento is a atypical RP subtype characterized by the absence of typical retinal pigment deposits while presenting with nyctalopia, visual field constriction, and abnormal electroretinography (ERG) findings (7). RPSP is often misdiagnosed as intermediate uveitis or other retinal disorders in the early stages due to its atypical presentation (8). Imaging modalities, such as optical coherence tomography (OCT), and functional tests, such as ERG, are critical for revealing structural and functional retinal damage and diagnosing (9). Although RPSP phenotypes have been documented, the genetic basis and mechanisms underlying the absence of bone spicule pigmentation remain poorly understood. Genes that have been rarely reported in association with RP, such as *GUCA1B* and *ABHD12*, play a role in the pathogenesis of RPSP (10, 11). These genes are functionally involved in phototransduction and lipid metabolism (12, 13). However, their specific roles in RPSP are yet to be elucidated.

To our knowledge, GUCA1B and ABHD12 have not been commonly associated with the RPSP phenotype; reporting these variants expands the genotypic and phenotypic spectrum of RPSP. This study aimed to analyze multimodal imaging and whole-exome sequencing (WES) data from two cases of RPSP to explore potential pathogenic mechanisms. The findings can help advance the understanding of RPSP’s molecular basis, genetic heterogeneity, and disease mechanisms and provide valuable insights into improving genetic counseling and early diagnosis.

## Materials and methods

### Patient recruitment and clinical evaluation

Two patients with clinical features suggestive of RPSP were enrolled from (Department of Ophthalmology, Tianjin Medical University General Hospital). This study was conducted in accordance with the Declaration of Helsinki and written informed consent was obtained from all participants or their legal guardians.

All participants underwent a comprehensive ophthalmic evaluation, including uncorrected visual acuity, best-corrected visual acuity (BCVA), slit-lamp biomicroscopy, and intraocular pressure (IOP) measurement using a non-contact tonometer (NIDEK NT-530P, Japan). Dilated fundus examination was performed using indirect ophthalmoscopy and a 90D lens.

### Multimodal retinal imaging

#### Multicolor and fundus autofluorescence (FAF) imaging

Multicolor scanning laser imaging and FAF were performed using the Mirante scanning laser ophthalmoscope (SLO) system (NIDEK, Japan). FAF was obtained with a 488 nm excitation wavelength and a 500–700 nm emission filter, and patterns of hyper- or hypoautofluorescence were recorded to assess RPE function.

#### Optical coherence tomography (OCT)

Macular microstructure was evaluated using spectral-domain optical coherence tomography (CIRRUS HD-OCT 5000, Carl Zeiss Meditec, Germany). A macular cube scan centered on the fovea was acquired for each eye. Retinal layer segmentation, cystoid changes, ellipsoid zone (EZ) integrity, and outer retinal atrophy were analyzed.

#### Adaptive optics scanning laser ophthalmoscopy (AOSLO)

Photoreceptor imaging was performed using the Mona II AOSLO system (Robotrak Technologies, China). High-resolution en face images of the central retina were acquired. Cone photoreceptor mosaic was evaluated for density, spacing, and structural integrity. Regions of cone loss or signal dropout were assessed qualitatively and quantitatively.

#### Fluorescein angiography (FA)

Fluorescein angiography was performed with the Heidelberg Spectralis HRA (Heidelberg Engineering, Germany) following intravenous injection of 5 mL of 10% fluorescein sodium. Early and late-phase images were captured to identify areas of RPE window defects, leakage, and macular edema.

### Visual field testing

Static perimetry was conducted using the Octopus 900 perimeter (Haag-Streit, Switzerland), employing a central 30° grid. Visual defect and mean deviation (MD) were documented and compared with normative data.

#### Visual electrophysiological assessments

Full-field electroretinography (ffERG) and pattern visual evoked potentials (PVEP) were performed at external tertiary ophthalmology centers, following the standards of the International Society for Clinical Electrophysiology of Vision (ISCEV). The ffERG included scotopic 0.01, scotopic 3.0, oscillatory potentials, photopic 3.0, and 30-Hz flicker responses. PVEP recordings were obtained using 0.5 cycles per degree (cpd) checkerboard stimuli, and the latencies and amplitudes of the N75, P100, and N135 components were analyzed for each eye (14, 15).

#### Genetic testing and bioinformatics analysis

Whole-exome sequencing was performed for both probands by certified commercial laboratories. For Patient 1, WES was conducted by MyGenostics Inc. (迈基诺, Beijing, China); for Patient 2, sequencing and primary analysis were performed by BGI Genomics (华大基因, Shenzhen, China). Genomic DNA was extracted from peripheral blood using standard protocols, and sequencing was carried out on the Illumina HiSeq platform with a mean coverage of ≥100× (16).

Sequencing reads were aligned to the human reference genome (GRCh37/hg19), and variant calling was performed using the BWA-GATK pipeline. Population-level filtering included gnomAD (r2.1.1) (all populations and the East Asian subset), ExAC (r1) (East Asian subset), 1000 Genomes (Phase3), and dbSNP (2.9.1). We additionally checked ExAC-EAS (∼4,000 East Asian individuals) and gnomAD-EAS to confirm absence of the reported variants in East Asian cohorts (see Results for dataset findings). Pathogenicity of missense variants was predicted using multiple *in silico* tools, including:

- SIFT v5.2.2 (deleterious if score < 0.05) (17),
- PolyPhen-2 v2.2.2 (probably damaging if score > 0.85) (18),
- MutationTaster (version 2)^1^ (disease-causing prediction) (19).
- Revel v1.3 (likely benign if score < 0.3, likely pathogenic if score > 0.5) (20).

Variants were interpreted and classified according to the American College of Medical Genetics and Genomics (ACMG/AMP2015) guidelines (21). Segregation analysis in available family members was performed via Sanger sequencing.

## Results

### Patient 1

A 39-years-old woman presented with decreased visual acuity (VA) and metamorphopsia in her left eye (OS), accompanied by night vision impairment without obvious nyctalopia. She had no history of ocular trauma or surgery, and no known systemic comorbidities were reported. Her baseline VA was 20/20 in the right eye (OD) and 20/63 in the OS, with normal intraocular pressures. Slit-lamp examination revealed no anterior segment abnormalities. Fundus biomicroscopy revealed bilateral macular pallor.

**FIGURE 1:**
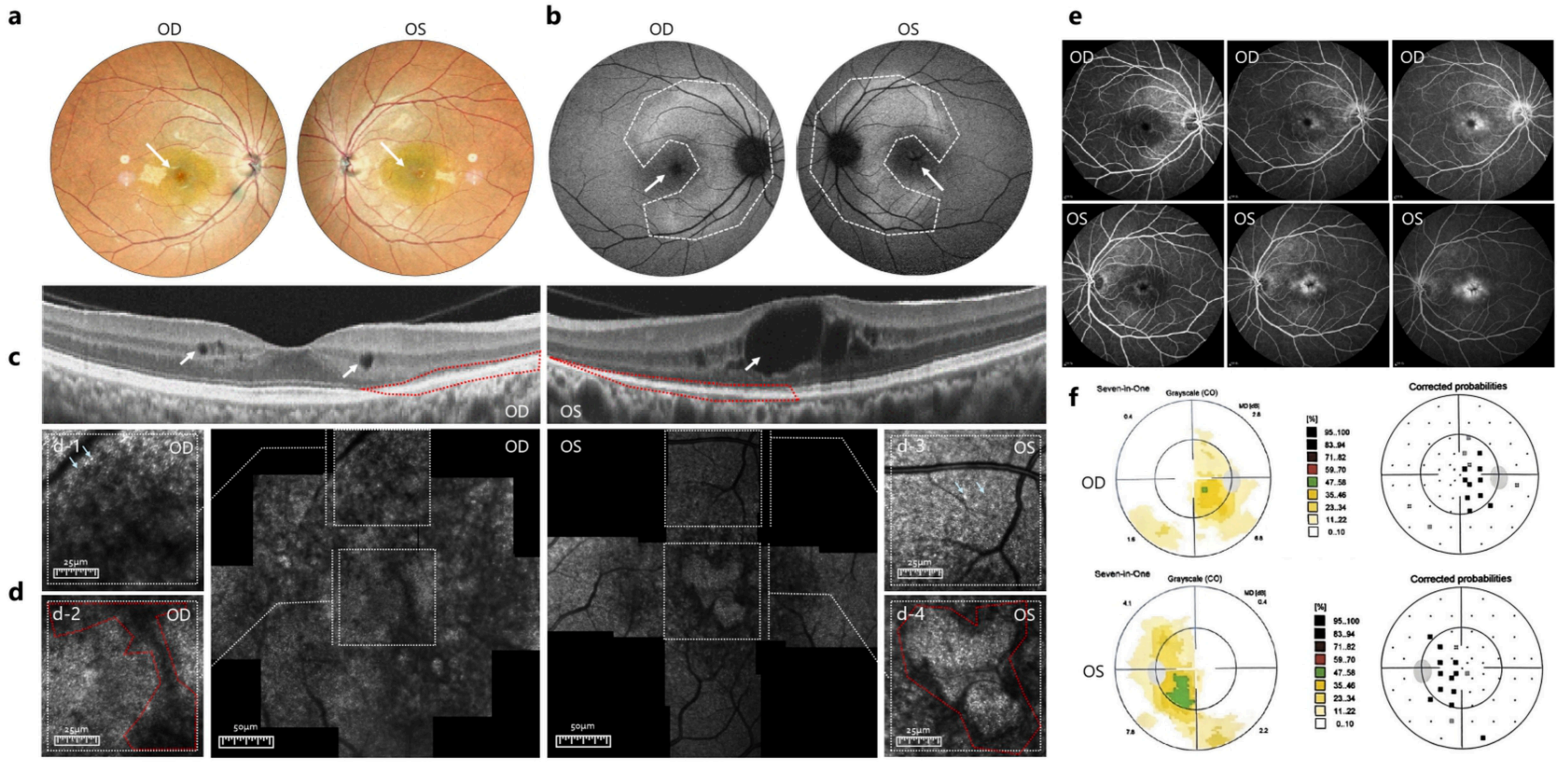
Multimodal ophthalmic imaging of Patient 1. **(a)** Multicolor scanning laser fundus imaging of both eyes showed macular edema (indicated by arrow). **(b)** FAF showed patchy hyperautofluorescence (highlighted in the dotted line) and central hypoautofluorescence (indicated by arrow). **(c)** OCT of the macula showed cysts in INL (indicated by white circle), EZ disruption and perifoveal outer retinal atrophy (highlighted in the dotted red line). **(d)** AOSLO images heterogeneous photoreceptor cells size with relatively preserved morphology and increased inter-cone spacing (indicated by arrow in d-1 and d-3) and multiple hyporeflective areas in macula (highlighted in the dotted red line in d-2), in OS “isolated” photoreceptor clusters obscured by shadowing (highlighted in the dotted red line in d-4). **(e)** FA of both eyes showed RPE window defects and macular leakage. **(f)** Central visual field revealed bilateral central scotomas.

**FIGURE 2:**
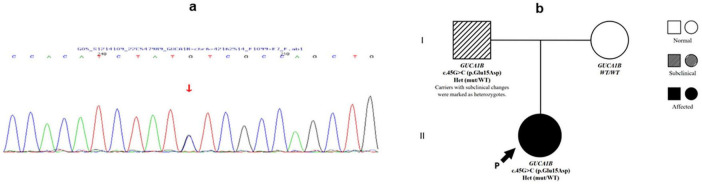
Genetic findings of Patient 1. **(a)** Sanger sequencing chromatogram of Patient 1 showing a heterozygous c.45G > C variant in the GUCA1B gene (indicated by arrow). **(b)** Pedigree of Patient 1’s family demonstrating autosomal dominant inheritance. The proband (arrow) and her affected father carry the heterozygous GUCA1B c.45G > C mutation, while the mother is unaffected and wild-type.

**FIGURE 3:**
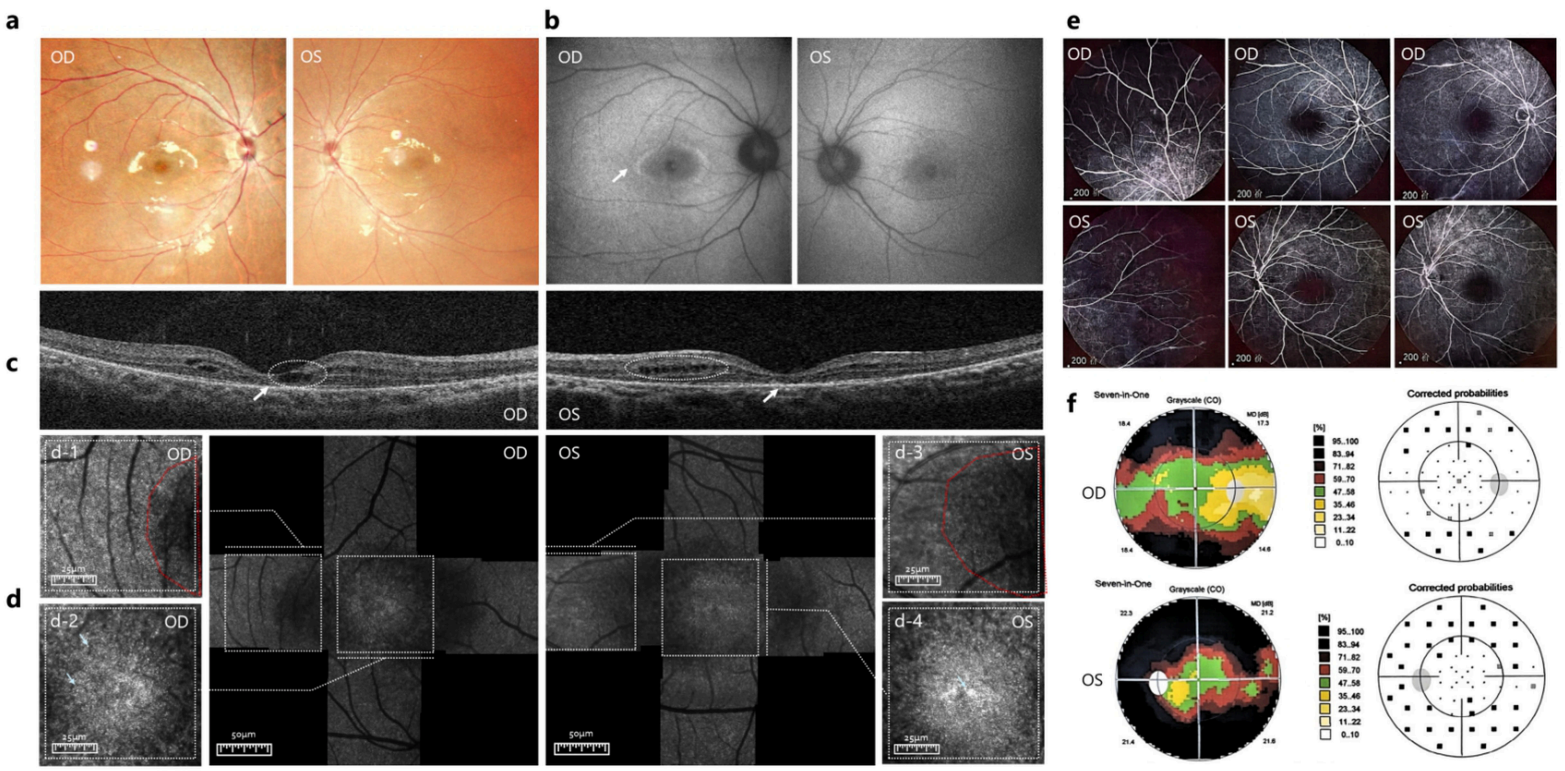
Multimodal retinal imaging of Patient 2. **(a)** Multicolor scanning laser fundus imaging of both eyes showed the absence of bone spicule pigmentation. **(b)** FAF showed macular hypoautofluorescence and hyperautofluorescent perimacular ring (indicated by arrow in OD). **(c)** OCT of the macula showed intraretinal cysts (indicated by white dotted circle), EZ disruption, and outer retinal atrophy (indicated by arrow). **(d)** AOSLO images disorganized photoreceptor mosaics at the fovea with numerous hyperreflective deposits (indicated by arrow in d-2 and d-4) extensive hyporeflective areas and shadowing artifacts with no observation of cone across the macula (highlighted in the dotted red line in d-1 and d-3). **(e)** FA of both eyes midperipheral window defects, widespread peripheral vascular leakage, and enlargement of the foveal avascular zone. **(f)** Central visual field revealed extensive superior and inferior defects in the OD and tubular constriction in the OS.

**FIGURE 4:**
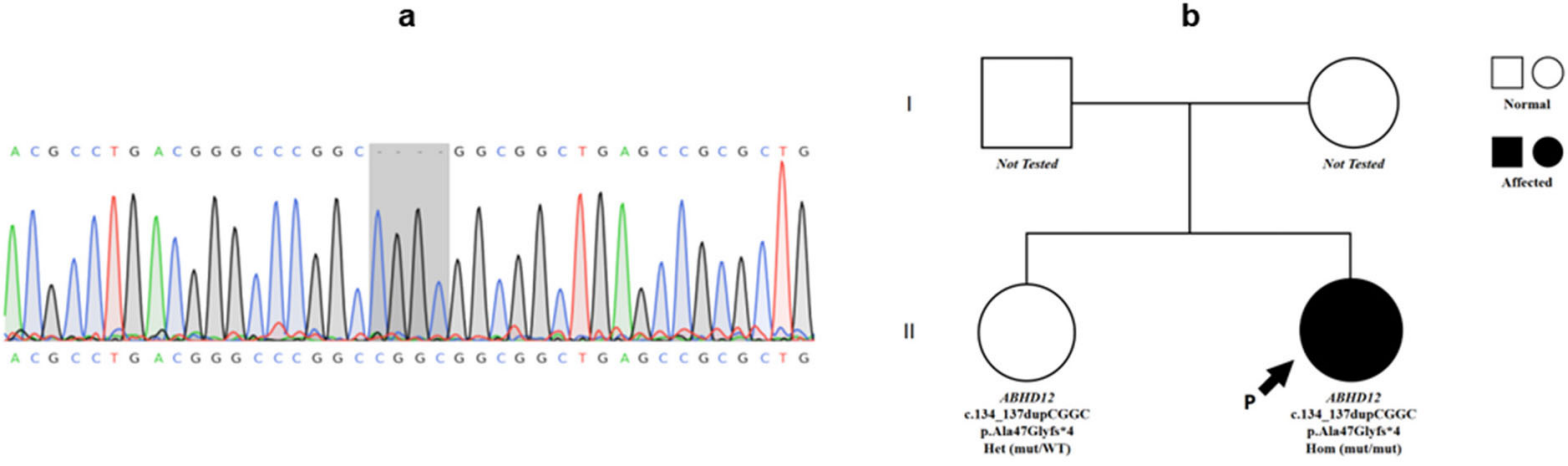
Genetic findings of Patient 2. **(a)** Sanger sequencing chromatogram showing a homozygous frameshift mutation in ABHD12 (c.134_137dupCGGC, p.Ala47Glyfs*4) in Patient 2 (highlighted in the gray box). **(b)** Pedigree of Patient 2’s family. The proband (arrow) is homozygous for the ABHD12 mutation. Her sister is heterozygous, while parental genotypes were not available.

Multicolor scanning laser fundus imaging showed no bone spicule pigmentation but showed macular edema (Figure 1a). FAF imaging revealed patchy hyperautofluorescence in the midperipheral retina, a perivascular hyperautofluorescent ring, and macular hypoautofluorescence, indicative of RPE dysfunction or photoreceptor loss (Figure 1b). OCT of the OD revealed small intraretinal cysts in the inner nuclear layer (INL), EZ disruption, and outer retinal atrophy around the fovea. Marked macular elevation, large INL cysts, EZ disruption, and perifoveal outer retinal atrophy were observed in the OS (Figure 1c). AOSLO imaging of the OU revealed a heterogeneous photoreceptor cell morphology with increased inter-cone spacing (Figure 1d-1, 3), imaging of the OD showed multiple hyporeflective areas (Figure 1d-2), indicative of photoreceptor loss or shadowing by edema. AOSLO imaging of the OS revealed extensive hyporeflective zones (photoreceptor loss) and “isolated” photoreceptor clusters obscured by shadowing (Figure 1d-4). FA showed RPE window defects and macular leakage (Figure 1e).

Central visual field testing revealed bilateral central scotomas, which were more pronounced in the OS (Figure 1f). ERG showed a mildly reduced maximal combined response and rod oscillatory potentials and moderately reduced cone amplitudes with preserved 30 Hz flicker in the OD and moderately reduced rod oscillatory potentials with mildly diminished 30 Hz flicker and severely reduced cone amplitudes in the OS.

The patient’s father reported occasional minor collisions while driving. His BCVA was 20/20. Multicolor scanning laser fundus imaging revealed no bone-spicule–like pigmentation and FAF showed patchy hyperautofluorescence in the mid-peripheral retina. Macular OCT was generally normal, but peripheral OCT demonstrated areas of RPE atrophy. Visual field testing indicated bilateral nasal scotomas. Although he reported no subjective visual symptoms, these findings suggested subclinical retinal impairment.

Whole-exome sequencing identified a heterozygous c.45G > C (p.Glu15Asp) missense mutation in *GUCA1B* (Figure 2). Bioinformatic predictions for this variant showed conflicting results: SIFT (benign), PolyPhen-2 (benign), MutationTaster (disease-causing), GERP+ (disease-causing), and REVEL meta-predictor (benign). Following ACMG/AMP2015 guidelines (20), we classified this variant as of uncertain significance (VUS) with conflicting criteria (PM2_Supporting: absent in population databases; BP4_Moderate: multiple benign *in silico* predictions). This variant was not reported in ClinVar, HGMD, or gnomAD, and was absent in the ExAC-East Asian dataset (∼4000 individuals), supporting its classification as a novel mutation.

Family segregation analysis revealed that the father carried the same mutation with subclinical retinal changes, indicating reduced penetrance or variable expressivity of GUCA1B-associate RPSP.

### Patient 2

A 22-years-old woman presented with progressive nyctalopia, concentric visual field constriction over 2 years, and binocular nystagmus, accompanied by a significant visual decline in the OD. She had no relevant ocular history or systemic comorbidities. Her family history revealed no symptomatic relatives, including her parents and an elder sister, and there was no known consanguinity between her parents. Her baseline VA was 20/50 in the OD and 20/80 in the OS, with normal intraocular pressures. Slit-lamp examination revealed unremarkable anterior segments. Fundus biomicroscopy revealed mildly tilted and hyperemic optic disks (cup-to-disk ratio of 0.3), attenuated retinal vessels, diffuse retinal graying, and macular edema with a perifoveal ring.

Multicolor scanning laser fundus imaging showed the absence of bone spicule pigmentation (Figure 3a). FAF demonstrated heterogeneous midperipheral autofluorescence intensity, macular hypoautofluorescence (indicative of RPE dysfunction), and a hyperautofluorescent perimacular ring in OD (indicative of increased RPE metabolic stress) (Figure 3b). Bilateral macular OCT revealed intraretinal cysts, EZ disruption, and outer retinal atrophy (Figure 3c). AOSLO imaging revealed disorganized photoreceptor mosaics at the fovea, with numerous hyperreflective deposits (Figure 3d-2, 4) (indicative of photoreceptor degeneration), extensive hyporeflective areas (Figure 3d-1, 3) (indicative of photoreceptor loss or microcysts), and shadowing artifacts across the macula. FA showed midperipheral window defects, widespread peripheral vascular leakage, and enlargement of the foveal avascular zone (Figure 3e).

Central visual field testing showed extensive superior and inferior defects in the OD (mean deviation: −17.2 dB) and tubular constriction in the OS (mean deviation: −21.5 dB) (Figure 3f). Visual evoked potentials showed prolonged P100 latencies bilaterally and reduced amplitude in the OS.

Whole-exome sequencing identified a homozygous frameshift mutation (c.134_137dupCGGC, p.Ala47Glyfs*4) in *ABHD12* (Figure 4). This mutation was predicted as Pathogenic according to ACMG/AMP (2015) criteria (PVS1: predicted null variant in a gene where loss-of-function is a known mechanism; PM2: absent from population databases; PM3_Supporting: segregation/allelic data supporting recessive inheritance). The ABHD12 c.134_137dupCGGC (p.Ala47Glyfs*4) frameshift variant was not present in ClinVar, HGMD, or dbSNP, and was absent in gnomAD-East Asian populations further supporting its classification as a novel pathogenic mutation.

Family segregation revealed that the asymptomatic sister carried the heterozygous variant, consistent with an autosomal recessive inheritance pattern.

## Discussion

### Imaging features of RPSP

Retinitis pigmentosa sine pigmento is clinically defined by nyctalopia, visual field constriction, and absence of characteristic bone spicule pigmentation on fundus examination (7). Multimodal imaging techniques, particularly OCT and FAF, play pivotal roles in RPSP diagnosis and differential evaluation.

Optical coherence tomography enables precise visualization of the retinal laminar architecture, revealing early macular abnormalities in RPSP. After photoreceptor loss, compensatory Müller cell stress responses may contribute to INL edema (22). Impaired clearance of photoreceptor metabolic waste by dysfunctional RPE can lead to debris accumulation between the ONL and INL (23). Such deposits may disrupt Müller cell homeostasis and compromise the blood-retinal barrier (BRB), potentially worsening fluid leakage and intraretinal edema (24).

Fundus autofluorescence is an essential tool for assessing RPE functional status. Both patients exhibited macular hypoautofluorescence, indicating regional RPE dysfunction (Figures 1b, 2b) (25). A hyperautofluorescent perimacular ring, which is a recognized imaging hallmark of RPSP, was observed, likely demarcating the transition zone between the degenerating and relatively preserved photoreceptors (26). This annular pattern may contract centrally during disease progression, correlate with visual field deterioration, and serve as a potential progression biomarker (27).

Adaptive optics scanning laser ophthalmoscopy imaging enables cellular-level resolution assessment of retinal microstructure, revealing degenerative changes in photoreceptor cells. The AOSLO findings predominantly revealed reduced cone density, disorganized cellular arrangement, and hyperreflective foci in patients with RPSP. These hyperreflective deposits are associated with accumulated metabolic byproducts from photoreceptor outer segments and RPE cells, representing residual photoreceptor structures or degenerative cellular debris (28), including lipofuscin aggregates and calcified deposits (29). In Patient 1, heterogeneous cone spacing at the foveal center with preserved photoreceptor morphology indicated relatively mild macular damage, consistent with minimally impaired BCVA. Conversely, in Patient 2, extensive hyperreflective material which indicate severe photoreceptor loss, with only isolated intact photoreceptor clusters, significantly correlated with reduced BCVA.

Electroretinography is a critical functional assessment of photoreceptor activity. In Patient 1, this pattern aligns with the phototransduction defects caused by the *GUCA1B* mutation (c.45G > C), which disrupts the calcium-dependent regulation of retinal cyclic nucleotide signaling. These findings are consistent with previous reports of similar ERG abnormalities in patients with RPSP (30), highlighting the diagnostic utility of ERG in characterizing photoreceptor dysfunction in atypical RP variants.

In summary, multimodal imaging examinations play a crucial role in the early diagnosis of RPSP: thinning of the ONL and IS/OS observed on OCT are sensitive early indicators, while macular edema may reflect secondary vascular leakage. Hypoautofluorescent areas on FAF are associated with dysfunction of the RPE’s phagocytic activity, aiding in the differentiation between RPSP and inflammatory diseases. On AOSLO, findings such as reduced cone cell density, disorganized cellular arrangement, and the appearance of hyper-reflective dots suggest photoreceptor damage, providing a significant advantage in detecting early-stage RPSP. Despite the absence of pigmentary deposits, these features hold critical diagnostic and differential diagnostic value, whereas WES facilitates precise identification of the causative mutations.

### Genetic mutations associated with RPSP

Retinitis pigmentosa sine pigmento is characterized by marked genetic heterogeneity, with only a limited number of genes currently implicated in its pathogenesis. Table 1 lists the reported genes associated with RPSP, their chromosomal loci, functional roles, and inheritance patterns.

**TABLE 1:** Genes associated with RPSP.

Gene	Chromosomal locus	Function	Inheritance pattern
*GUCA1B* (51)	6p21.1	Calcium ion binding and signaling	Autosomal dominant
*ABHD12* (42)	20p11.21	Lipid metabolism and neuroprotection	Autosomal recessive
*RPGR* (52)	Xp11.4	Photoreceptor cilia maintenance	X-linked
*RHO* (53)	3q22.1	Rod phototransduction	Autosomal dominant/autosomal recessive
*PRPH2* (54)	6p21.1	Photoreceptor outer segment structure	Autosomal dominant/digenic

Emerging evidence links *GUCA1B* and *ABHD12* mutations to RPSP pathogenesis. Although these variants underlie other RP subtypes, further functional validation and clinical corroboration are required to elucidate their specific roles in RPSP.

### Pathogenic mechanisms of GUCA1B mutations

The *GUCA1B* gene encodes guanylyl cyclase-activating protein 2 (GCAP2), a critical regulator of retinal phototransduction. GCAP2 modulates guanylyl cyclase activity via calcium-dependent interactions, thereby maintaining photoreceptor light adaptation (31, 32).

The c.45G > C (p.Glu15Asp) mutation resides in exon 1 of *GUCA1B* and is localized to the N-terminal non-EF-hand domain (amino acid 15) distal to its three EF-hand motifs (EF1: 25–56; EF2: 65–96; EF3: 105–136) (12). Although the N-terminal domain may stabilize protein conformation or mediate interactions with signaling partners, its precise functional role remains uncharacterized.

Classical *GUCA1B* mutations affecting EF-hand domains typically manifest as pigmented RP with bone spicule deposits (33). Conversely, our N-terminal mutation (p.Glu15Asp) was associated with RPSP and macular edema, potentially due to the following:

1. Preserved RPE phagocytic function: N-terminal mutations may spare RPE metabolic activity, reducing lipofuscin accumulation and pigment deposition (34).
2. Inflammatory/vascular mechanisms: Previous studies indicate that altered GCAP2 activity disrupts retinal homeostasis, fostering a pro-inflammatory state with upregulated VEGF and other permeability mediators (35). Elevated VEGF, in turn, is a well-established driver of BRB breakdown and macular edema in retinal degenerations (36). Thus, the mutation may increase VEGF expression, contributing to BRB breakdown and aggravating macular edema (24).
3. Incomplete penetrance: The proband’s father carried the same mutation with subclinical retinal changes but intact BCVA, indicating modulation by genetic/environmental factors (37).

Although the N-terminal mutation does not directly impair calcium binding, it may drive pathology through the following:

1. Structural destabilization: The p.Glu15Asp substitution (negative charge → neutral) can perturb the tertiary structure of GCAP2, impairing the guanylyl cyclase interaction (12, 38).
2. Subcellular mislocalization: Altered N-terminal signaling may disrupt GCAP2 trafficking to the outer segments of photoreceptors, thereby compromising signal transduction (39).
3. Non-canonical pathways: The N-terminal domain may regulate oxidative stress and autophagy pathways. Its dysfunction can indirectly trigger photoreceptor apoptosis (40).

For therapeutic implications, EF-hand domain mutations may respond to calcium homeostasis modulators, whereas N-terminal variants, such as p.Glu15Asp, may require targeted anti-inflammatory or anti-VEGF agents to alleviate vascular leakage (41).

### Pathogenic mechanisms of *ABHD12* mutations

The *ABHD12* gene (20p11.21) encodes α/β-hydrolase domain-containing protein 12 (ABHD12), a serine hydrolase critical for hydrolyzing lysophosphatidylserine (lyso-PS) to maintain lipid homeostasis in the central nervous system and retina (42).

The homozygous c.134_137dupCGGC (p.Ala47Glyfs*4) frameshift mutation identified in Patient 2 disrupts the N-terminal transmembrane domain of ABHD12, resulting in a premature stop codon (truncated to 51 amino acids). This mutation is predicted to undergo nonsense-mediated mRNA decay, leading to the complete loss of the ABHD12 protein (43). ABHD12 deficiency leads to pathological lyso-PS accumulation in retinal and neural tissues, triggering microglial activation and proinflammatory cytokine release (e.g., TNF-α and IL-6), which drive chronic inflammation and photoreceptor apoptosis (44). Lyso-PS overload upregulates VEGF expression, compromises BRB integrity, and induces macular edema via vascular leakage (45). Furthermore, aberrant lipid metabolism impairs mitochondrial function, worsening photoreceptor energy deficits (46).

Although *ABHD12* mutations are classically associated with PHARC syndrome, such as polyneuropathy, hearing loss, ataxia, retinopathy, and cataracts (42), Patient 2 exhibited binocular nystagmus but showed no additional neurological or auditory manifestations. The absence of systemic features in this case may be explained by several factors:

1. Age of onset and disease progression: PHARC is typically early-onset and progressive, and systemic manifestations may appear later in life. Thus in our case, extra-ocular features might not yet have manifested (13).
2. Incomplete penetrance and variable expressivity: Among reported PHARC patients, many do not present all five characteristic features; severity and symptom combinations vary considerably, perhaps due to genetic modifiers or environmental factors (13).
3. Non-syndromic or mild phenotypes: Cases have been documented in which ABHD12 mutation carriers exhibit retinal degeneration (RP) but lack overt neuropathy, ataxia, or hearing loss even after detailed systemic evaluation (47).
4. Tissue-specific effects: The N-terminal truncation (p.Ala47Glyfs*4) may predominantly impair retinal ABHD12 function, with compensatory mechanisms, such as ABHD6 activity, preserving systemic lipid metabolism (48).
5. Localized inflammation: Lyso-PS accumulation may preferentially activate retinal microglia without triggering systemic inflammation (45).

The lack of bone spicule pigmentation in this case can be attributed to the following reasons:

1. Preserved RPE phagocytosis: If ABHD12 deficiency spares RPE phagocytic pathways, such as MER-TK receptor signaling or lysosomal degradation, reduced lipofuscin accumulation may explain pigment deposit absence (49).
2. Non-inflammatory apoptosis: N-terminal truncations may induce photoreceptor death via non-inflammatory pathways, such as endoplasmic reticulum stress, minimizing RPE activation and pigment migration (50).
3. Phenotypic masking: Early-onset macular edema in N-terminal mutations (vs. catalytic domain variants, such as p.Arg349Gln) can obscure the pigmentary changes typically observed in later disease stages (13).

## Conclusion

This study identified two novel mutations, *GUCA1B* c.45G > C and *ABHD12* c.134_137dupCGGC, in patients with RPSP, supported by multimodal imaging and functional assessments consistent with the RPSP diagnostic criteria. This study further highlights the critical role of multimodal imaging in early diagnosis of retinitis pigmentosa sine pigmento (RPSP). FAF enables functional assessment of RPE, while OCT detects microstructural retinal alterations. AOSLO reveals early photoreceptor damage. When combined with functional testing and genetic analysis, this integrated approach significantly improves diagnostic accuracy in early-stage RPSP. Further studies that prioritize the functional validation of these mutations in model systems and explore targeted therapeutic strategies tailored to their distinct pathogenic mechanisms are needed.

## Data Availability

The original contributions presented in the study are publicly available. This data can be found at the ClinVar database under accession numbers SCV006336833 and SCV006336834.

## Footnotes

1
http://www.mutationtaster.org/
